# Use of Pharmacotherapies in the Treatment of Alcohol Use Disorders and Opioid Dependence in Primary Care

**DOI:** 10.1155/2015/137020

**Published:** 2015-01-05

**Authors:** Jinhee Lee, Thomas F. Kresina, Melinda Campopiano, Robert Lubran, H. Westley Clark

**Affiliations:** ^1^Center for Substance Abuse Treatment, Substance Abuse and Mental Health Services Administration, Rockville, MD 20857, USA; ^2^Division of Pharmacologic Therapies, Center for Substance Abuse Treatment, Substance Abuse and Mental Health Services Administration, 1 Choke Cherry Road, Rockville, MD 20857, USA

## Abstract

Substance-related and addictive disorders are chronic relapsing conditions that substantially impact public health. Effective treatments for these disorders require addressing substance use/dependence comprehensively as well as other associated comorbidities. Comprehensive addressing of substance use in a medical setting involves screening for substance use, addressing substance use directly with the patient, and formulating an appropriate intervention. For alcohol dependence and opioid dependence, pharmacotherapies are available that are safe and effective when utilized in a comprehensive treatment paradigm, such as medication assisted treatment. In primary care, substance use disorders involving alcohol, illicit opioids, and prescription opioid abuse are common among patients who seek primary care services. Primary care providers report low levels of preparedness and confidence in identifying substance-related and addictive disorders and providing appropriate care and treatment. However, new models of service delivery in primary care for individuals with substance-related and addictive disorders are being developed to promote screening, care and treatment, and relapse prevention. The education and training of primary care providers utilizing approved medications for the treatment of alcohol use disorders and opioid dependence in a primary care setting would have important public health impact and reduce the burden of alcohol abuse and opioid dependence.

## 1. Introduction

Substance-related and addictive disorders are chronic conditions estimated to occur in one in five patients in primary care [1, 2]. Primary care is an important entry point for all patients suffering from chronic conditions. Primary care health providers are the best positioned to address substance-related and addictive disorders in a comprehensive manner encompassing screening, prevention, diagnosis, disease management, and relapse prevention [3, 4]. However, currently in primary care, there is poor adoption of pharmacotherapies that have demonstrated effectiveness for alcohol use disorders [5, 6]. In addition, there is a national public health crisis related to opioid misuse and abuse that has a high impact on the health care system that requires primary health care providers to assume an even greater important role in providing evidence-based effective prevention, care, and treatment [7].

At present, primary care patients who are in need of treatment and willing to access treatment are referred to specialty care providers via a process which can seem obscure with barriers and challenges to patient care coordination and follow-up. In this referral system, the patient is often lost in the gap between primary care and specialty treatment systems and fails to engage in care and treatment successfully. Opioid-dependent patients who require and choose pharmacotherapy treatment, such as medication assisted treatment, may find that treatment capacity in their community is inadequate, effectively denying them assess [8, 9]. Consequently patients may undergo detoxification and not enter managed chronic care or may experience a period of psychosocially based treatment after which relapse to substance use is common or, alternatively, fail to be retained in chronic care and treatment due to the unmet need for medication.

## 2. Addressing Substance-Related and Addictive Disorders in Primary Care through Screening, Brief Interventions, and Treatment

The US Preventive Task Force recommends screening all patients for alcohol misuse and addressing hazardous or harmful use through a brief intervention [10]. Screening instruments and evaluation protocols are important initial tools needed in addressing alcohol use/dependence and drug abuse [2, 3, 11, 12]. Screening instruments such as the AUDIT, ASSIST, CAGE questionnaire, or other validated tool can be utilized to determine hazardous or harmful alcohol use, as well as identifying drug abuse and opioid dependence to help inform the primary care provider in determining the level of care and treatment setting for the patient [13–15]. Screening and brief interventions to reduce alcohol use have been shown to be both acceptable to patients and effective in primary care, but barriers do exist [14–16]. For primary health care providers trained in screening, brief interventions, and referral to treatment, these barriers include lack of organizational support, lack of physician time, and inconsistent communication [16]. Training of primary health care providers in screening and brief interventions is most accepted using web-based training programs; however, curriculum-based traditional training programs of residents have been well received [17, 18]. Evidence supporting the application of universal screening and brief intervention for drug use is lacking [19, 20]. However, screening and brief intervention of adolescents, a time early in the use of illicit drugs such as cannabis, has shown some effectiveness [21, 22]. Keeping in view the high prevalence and associated morbidity and mortality of chronic drug and alcohol dependence [16], the impact of unidentified substance-related and addictive disorders on the management of comorbid conditions such as diabetes, heart disease, and liver disease [23], the natural history of substance-related and addictive disorders [4], and the availability of effective pharmacotherapies, the primary care health professional may give serious consideration to applying the same or similar screening and brief intervention strategies to all substance-related and addictive disorders. It would be good clinical practice, from the point of view of efficiency and utility, to implement and maintain a screening and brief intervention process that is comprehensive addressing not only alcohol use disorders, but also tobacco and other substances use and misuse [17].

### 2.1. Patient Assessment and Referral out of Primary Care to Specialty Care

Screening and brief intervention for harmful or hazardous alcohol use is an important clinical tool for primary care providers to utilize and successfully deliver in their clinical practice. Beyond training and delivery of a brief intervention, an important component of primary care is the assessment of the patient and subsequent diagnosis of alcohol or drug dependence. Brief interventions may not be efficacious for individuals who are heavy drinkers, alcohol-dependent, or who have a severe co-occurring mental health problem [16, 24]. For those co-occurring disorder patients, providing screening and brief interventions through a trained psychologist may be a preferable clinical option. The option for the primary care provider is to assess which type of care, primary or referral specialty addiction treatment will be most beneficial to the patient. This assessment should also determine the patient's need, motivation for and choice of treatment as well as establishing a baseline against which patient response to and choice of treatment (pharmacotherapy) or disease progression can be measured. This assessment should also note the likelihood of relapse during chronic management. While these complex issues might suggest a better clinical outcome from specially addiction treatment, a recent clinical trial of alcohol care management delivery in primary care compared to specialty addiction treatment suggests otherwise [25]. The results of this randomized clinical trial suggest that providing intensive care and pharmacotherapy in a primary care setting provides better clinical outcomes for patients with alcohol use disorders than those obtained in addiction specialty care. Thus, providing alcohol care management, including pharmacotherapy in primary care, can be an alternative to brief intervention and referral to addiction specialty care.

For opioid-dependent patients, the option for the primary care provider and patient is pharmacotherapy in primary care with either buprenorphine (partial opioid agonist) or naltrexone (opioid antagonist) or referral to an opioid treatment program where methadone (opioid agonist) or other controlled medications are dispensed under federal regulation [26]. An important consideration in this assessment is the common occurrence of fatal opioid overdose by patients in maintenance treatment on relapse to illicit opioid use [27].

## 3. Pharmacotherapies for Substance-Related and Addictive Disorders

The role of medication assisted treatment, the provision of medications as part of comprehensive care, varies according to the needs and goals of the patient [28, 29]. Reduction in substance use, overdose prevention, withdrawal from dependence, relapse prevention, and maintenance are all legitimate goals potentially served by Food and Drug Administration (FDA) approved medications. Only pharmacotherapies indicated for use in addictive disorders are presented in Table 1. Off-label use of medications without an FDA approved indication for addictive disorders and the role of pharmacotherapy for cooccurring psychiatric and medical disorders associated with relapse are beyond the scope of this paper.

### 3.1. Medically Managed Withdrawal (Detoxification)

Medically managed withdrawal or detoxification may be a necessary first step in recovery for patients, who are physically dependent on alcohol, opioids, or sedative/hypnotics. Medically managed withdrawal serves to palliate otherwise intolerable withdrawal symptoms and reduce the risk of serious medical consequences. Inpatient medically managed withdrawal is appropriate for patients at risk for severe withdrawal, delirium tremens, or with significant medical and psychiatric comorbidities [3, 12, 30]. Institutional protocols can be developed to assist in determining inpatient versus outpatient detoxification [12, 30, 31]. Regardless of the setting, medically managed withdrawal is not by itself addiction treatment but rather a bridge to treatment. Relapse and even death from overdose are not uncommon for those patients who do not successfully transition to treatment and/or support program to address chronic relapse to drug and/or alcohol use or abuse.

### 3.2. Pharmacotherapy for Alcohol Use Disorders

Patients who report one or more heavy drinking days in the past year or who have, for example, an AUDIT score greater than 8 should receive further assessment beyond a screening in primary care [2, 10, 32, 33]. In determining the need for pharmacotherapy, consideration should be given to (a) the factors motivating a patient toward treatment, (b) the patient's stage of change, (c) the potential for relapse, (d) the severity of any concomitant medical and psychiatric problems, (e) the patient's ability to tolerate medications, and (f) whether the patient is pregnant. If a patient engages in heavy drinking but does not meet the criteria for an alcohol use disorder, or meets only the criteria for mild alcohol use disorder, the clinician should use his or her professional judgment in helping the patient decide whether reducing or abstaining from alcohol is the more appropriate goal, based on factors such as a family history of alcohol problems and the patient's age or history of traumatic injuries related to drinking [33]. Information from family members and significant others can provide useful perspectives on the patient's status, as can communication with or records from clinicians who treated the patient in the past [16, 34]. The provider and patient should mutually agree on an initial goal and be willing to refine and revise that goal as treatment progresses [2]. For example, in working with a patient who is unwilling to set a goal of complete abstinence, the clinician should support the patient in reducing his or her drinking as an interim goal, while maintaining that complete abstinence is the safer strategy, with a greater chance of long-term success [10, 16, 35].

#### 3.2.1. Disulfiram

Disulfiram, the initial medication approved by the FDA for the treatment of alcohol dependence, is an alcohol aversive or alcohol sensitizing agent [5]. As such, disulfiram causes an acutely toxic physical reaction approximately 10–30 minutes after ingestion of alcohol. The reaction to alcohol can be multisystem comprising warmth and flushing of the upper chest and face, hyperventilation, blurred vision, chest pain, tachycardia, vertigo, marked confusion, and weakness. The reaction is usually proportional to the amount of alcohol and disulfiram ingested. Disulfiram does not reduce the urge to consume alcohol but does provide motivation to not use alcohol.

Patients who are good candidates for treatment with disulfiram include those who are motivated for treatment and want to achieve abstinence, those who are medically appropriate, those who can receive supervised dosing, and those who are capable of understanding the consequences of drinking alcohol while taking disulfiram. It also may be an appropriate short-term therapy for a patient in recovery who anticipates being in a situation that may trigger craving for alcohol (such as a family holiday visit) and who requests an additional incentive to remain abstinent [2, 36, 37]. Steps in initiating treatment with disulfiram are as follows [2, 36, 37]. (1) Wait until the patient has abstained from alcohol for at least 12 hours and/or until the breath or blood alcohol level is zero. (2) Perform an electrocardiogram if clinically indicated (as in a patient with a history of heart disease). (3) Confirm the absence of allergy to disulfiram. Monitoring should include confirmation of abstinence with breath or blood alcohol tests if needed, liver function, and other tests as clinically indicated. Patient education and ongoing supervision, as well as contingency management intervention, promote the efficacy of disulfiram.

#### 3.2.2. Naltrexone

The low rate of retention and adherence encountered with oral naltrexone led to the development of the extended-released injectable formulation, which was approved by FDA for the treatment of alcohol use disorders in 2006 [2, 38]. Oral naltrexone is most effective when prescribed for patients who are highly motivated and/or supported with observed daily dosing [39, 40]. Either form of naltrexone appears to be effective in the following patient populations: patients who have a history of opioid use disorder and who are seeking treatment for alcohol use disorder because naltrexone will reduce the reinforcing effects of and curb cravings for both opioids and alcohol; patients with intense craving for alcohol during treatment because they may experience greater medication benefit than patients with low levels of craving for alcohol; patients who have a family history of alcohol use disorders [38–41], both laboratory studies and clinical trials suggest that patients with a family history of alcohol problems may benefit more from treatment with naltrexone than patients who do not have such a history [42]; and patients with the Asp40 allele of the gene encoding the mu opioid receptor (OPRM1) [43]. Extended-release injectable naltrexone should be considered for patients who have problems with treatment adherence or do not have adequate support to comply with daily dosing [38].

Although patients may experience fewer medication side effects if they are abstinent from alcohol when they begin treatment with naltrexone, it is safe for patients to begin taking the medication during medically supervised withdrawal or if they are actively drinking [2, 44].

#### 3.2.3. Acamprosate

Acamprosate is an FDA approved medication for use in postwithdrawal maintenance of alcohol abstinence. Acamprosate normalizes the alcohol-related changes in the brain due to chronic alcohol consumption and reduces the symptoms of withdrawal, thereby reducing the potential to relapse to alcohol consumption [5]. A recent Cochrane review of 24 studies showed that acamprosate had a moderate effect on preventing a return to drinking and on increasing the number of days being abstinent [45]. Acamprosate appears to be most effective for patients who are motivated with a goal of complete abstinence as opposed to reduced consumption [46]. Thus, acamprosate can be utilized as a component of a treatment paradigm with the goal of maintenance of abstinence for individuals who are alcohol-dependent. Acamprosate typically is initiated five days after the cessation of alcohol use. However, it can be used safely in combination with alcohol (and benzodiazepines) and thus can be started during medically supervised withdrawal. The drug typically reaches full effectiveness in 5 to 8 days [2, 5, 36]. Acamprosate therapy should be continued even if a patient relapses to alcohol use [5]. Patients who may be particularly suited to treatment with acamprosate are those with hepatic disease; those who are being treated with opioids for pain or addiction because acamprosate is eliminated renally and does not affect endogenous or exogenous opioids; and those who are coping with multiple medical issues and who are taking many other medications because there are no clinically significant drug interactions with acamprosate.

## 4. Pharmacotherapy for Opioid Use Disorders

Any patient diagnosed with a moderate or severe opioid use disorder should be assessed for medication assisted treatment, the use of medications as part of a comprehensive treatment paradigm [29, 47]. Opioid agonists used in medication assisted treatment for opioid dependence, such as methadone and buprenorphine, can be used without prior detoxification. Extended-release injectable naltrexone is approved for relapse prevention and can be administered 7 to 10 days after last opioid use.

### 4.1. Methadone

Methadone, an opioid agonist, is a safe and effective pharmacologic therapy for the treatment of opioid use disorders [47, 48]. Recently, with the explosion of prescription opioid abuse and dependence in the United States, methadone treatment has been used successfully by opioid treatment programs (OTPs) for the treatment of prescription opioid abuse [49]. Methadone is a synthetic mu opioid receptor agonist with pharmacological properties qualitatively similar to morphine and was originally used to treat the painful symptoms of withdrawal from heroin and other opioids [50, 51]. Administered daily as an oral dose for the treatment of opioid dependence, an individualized therapeutic dosage of methadone is determined to maintain an asymptomatic state and stabilize a patient, without episodes of opioid overmedication or withdrawal. Minimum retention time in treatment varies for residential and outpatient methadone treatment programs. The National Institutes of Health consensus panel [52] concluded that individuals treated for fewer than three months with methadone do not show substantial medical gain. Methadone is usually the least expensive medication and when used in evidence-based treatment paradigms is cost effective and can result in reduced drug use, improved health outcomes, as well as improvements in quality of life [47, 53, 54]. Relapse to opioid misuse and abuse is common when methadone is discontinued without further support or behavioral treatment.

In the United States, methadone for the treatment of opioid dependence can only be dispensed in specialty clinics called Opioid Treatment Programs (OTPs). Studies have also shown that individuals receiving medication assisted treatment with methadone are at a reduced risk of death, half as likely to become infected with HIV, better able to comply with other medical therapies, less involved in crime, have an enhanced quality of life, with positive cognitive, emotional, and social functioning [55–58]. The federal regulations pertaining to the use of methadone for opioid use disorders do not preclude the integration of primary care into the specialty clinic [59]. Numerous models of integrated care and treatment have been piloted including studies showing the efficacy of transferring stable patients receiving mediation assisted treatment with methadone to primary care providers to continue treatment [60–62]. In such an arrangement where comprehensive medical care is provided with methadone maintenance treatment, it has been shown that ambulatory care increases with emergency room visits decreasing, resulting in cost-effective care [63].

### 4.2. Buprenorphine

Buprenorphine is a partial opioid agonist with a very high affinity for the mu opioid receptor [64]. Because it is a partial agonist, the effect of buprenorphine cannot be increased by taking larger amounts. This makes the drug less reinforcing, giving it less value as a drug of abuse and a favorable safety profile. The high receptor affinity creates a blockade effect so that, when dosed properly, the euphoric effects of other opioids are blunted or blocked. However, the abuse of other substances, such as benzodiazepines, may enhance respiratory depression and remains a contraindication with the use of opioid agonists. The combination of the partial agonist and high receptor affinity gives buprenorphine another unique property. It will cause an acute precipitated withdrawal if ingested in the presence of most full-agonist opioids. This also serves to reduce the abuse potential of buprenorphine. As an additional deterrent, buprenorphine has been formulated to include naloxone. Buprenorphine has poor oral bioavailability and moderate sublingual bioavailability. Therefore, formulations for opioid addiction treatment are in the form of sublingual tablets [65]. Naloxone, however, is not. The patient who takes buprenorphine combined with naloxone as directed will experience only the buprenorphine. However, if injected, the naloxone is fully effective and immediate withdrawal is experienced. Depot and implantable formulations are in development.

Buprenorphine has been shown to be safe and effective in the treatment of both injection (heroin) opioid use and prescription drug abuse and dependence [66, 67]. A recent study has shown that in a primary care setting using buprenorphine, prescription opioid-dependent patients showed better clinical outcomes compared to patients who were dependent on heroin [68]. Also, retention in buprenorphine maintenance treatment has been shown to reduce emergency department use, thus lowering the overall cost of care [69].

Buprenorphine can be prescribed, within certain parameters, by physicians in any medical practice. To do so, a physician must hold a current state medical license, a valid DEA registration number, and meet one or more of the following training requirements: hold a subspecialty board certification in addiction psychiatry from the American Board of Medical Specialties, hold a subspecialty board certification in Addiction Medicine from the American Osteopathic Association, hold an addiction certification from the American Society of Addiction Medicine, or have completed not less than 8 hours of authorized training on the treatment or management of opioid-dependent patients. This training may include classroom situations, seminars at professional society meetings, electronic communications, or other media. An office-based setting provides increased access to medication assisted treatment for opioid dependence in a less stigmatized environment and enables integrated primary medical care with the treatment of substance use disorders [70]. Medication assisted treatment with buprenorphine is reviewed in SAMHSA Treatment Improvement Protocol 40 [64].

### 4.3. Naltrexone

Naltrexone is a long-acting, opioid antagonist that blocks the euphoric effects of opioids binding the* mu* opioid receptor [71]. Unlike opioid agonists, administration does not relieve withdrawal nor does it cause withdrawal upon discontinuation. Due to naltrexone's opioid antagonism, patients prescribed naltrexone for opioid dependence must abstain from opioids for a minimum of seven days prior to starting naltrexone treatment to avoid the precipitation of opioid withdrawal. Naltrexone is most effective when utilized subsequent to the medical detoxification from opioids. The effectiveness of naltrexone treatment depends upon patient motivation and a social support system that promotes medication adherence [72]. Because of the need for adherence support interventions, the most recent Cochrane review of oral naltrexone treatment for relapse prevention to opioid use commented that oral naltrexone has not been scientifically demonstrated to be superior to other forms of treatment for opioid dependence [73]. Extended-release injectable naltrexone (Vivitrol) addresses the generally poor compliance with oral naltrexone through a monthly injectable formulation. Increased medication adherence was shown in a recent Phase 3 clinical trial that confirmed safety and efficacy of extended-release injectable naltrexone in the prevention of relapse to heroin use in a cohort of injection drug users [74]. A higher retention in care and higher rates of opioid-free urine screens were observed along with a significant reduction in opioid craving compared to placebo. Currently, studies are underway to determine the most efficacious primary care model(s) for the use of extended-release injectable naltrexone in the treatment of relapse prevention to heroin use.

## 5. Pharmacotherapies in Overdose Prevention in Primary Care

Overdose prevention education and naloxone prescribing complement the strategies employed in primary health care. Direct provision of the opioid-agonist therapies described above provide the primary care health provider the greatest opportunity to reduce the morbidity and mortality associated with opioid use disorders in particular, but also for alcohol use disorders. However, primary care health providers have a unique opportunity to support the health and prevent mortality for patients with less clear cut need, lack of access, or other barriers to entering formal specialty addiction treatment.

Naloxone is an opioid antagonist that acts by displacing opiates from receptor sites in the brain and reverses respiratory depression, a common cause of overdose deaths [75]. Naloxone has not been shown to cause physical or psychological dependence or tolerance [76] nor has it been associated with increased drug use or risky behavior. Overdose due to opioids is typically a slow process taking place over the course of several hours during which the ability to administer naloxone and provide rescue breathing promptly is lifesaving [77]. It is important to acknowledge that naloxone reverses only the effects of opioids. In the context of overdose related to multiple substances including opioids, it may still be sufficient to restore adequate respiration. If administered to an individual who is unresponsive due to a medical emergency not related to opioid toxicity, naloxone will have no effect.

While naloxone is most typically administered intravenously in the hospital or by medically trained first responders, it can be safely and effectively administered intramuscularly to simplify use. Administration of the injectable form via a nasal adaptor is becoming a wider practice although there is not presently a compatible FDA approved nasal adaptor or formulation specifically for intranasal use. Products, however, are in development [77]. Recently a new autoinjector device, Evsio, was approved by the FDA. The product is handheld and when turned on provides verbal instruction to the user similar to automated defibrillators.

SAMHSA also recommends consideration be given to coprescribing naloxone to patients receiving opioid analgesics [77]. For example, potential candidates to receive naloxone are patients undergoing a transition from one opioid treatment regimen to another, for whom it is medically necessary to take other potential respiratory depressants concurrently to manage other medical conditions, or who have respiratory or other illnesses increasing their susceptibility to respiratory depression. In addition by using screening and brief intervention tools (SBIRT), patients not previously recognized to be at risk for overdose can be identified. In these cases, overdose prevention and naloxone prescription form the basis of an appropriate brief intervention.

Primary health care providers are uniquely placed to address risk for overdose due to relapse to opioid use. Awareness of an individual's history of substance use disorder affords the primary care provider the opportunity to assess the stability and durability of a patient's recovery. A review of sober social supports, engagement in self-help, intensity and frequency of craving, and strategies for coping with cravings and external triggers can bolster an individual's ability to sustain his or her recovery. In the case of opioids in particular, relapse can be deadly due to loss of tolerance. Also intense feelings of shame and failure that can accompany relapse making it likely that relapse will be concealed as long as possible. Respectful inquiry as a matter of course in primary health care can prevent or identify relapse early. In such cases, therapy with the opioid-antagonist, extended-release injectable naltrexone, or oral naltrexone can be initiated to support recovery prior to relapse to substance use or promptly upon reestablishing abstinence after a relapse; some individuals may require opioid detoxification first. Naltrexone would also be an appropriate consideration for individuals returning to primary care from inpatient rehabilitation or incarceration.

## 6. Relapse Prevention

Relapse to drug and/or alcohol use after detoxification is common without additional interventions, treatment, and support. Peer support groups, behavioral counseling, and pharmacotherapy combined offer the best course for relapse prevention [78]. For relapse prevention to alcohol use, naltrexone, acamprosate, and disulfiram have been utilized with varied results [36]. Disulfiram has been shown to be effective in the treatment of alcohol use disorders when administered as a supervised low-dose disulfiram integrated with behavioral counseling and support groups [37]. Oral naltrexone has been shown to be effective in reducing heavy drinking days as well as with highly motivated patients or patients who have medication support structures; extended-release naltrexone, in combination with counseling and peer support, reduced the level of drinking from four drinks per day at baseline to less than one drink per day within three months in a primary care setting [39]. Follow-up studies showed that extended-release naltrexone can promote lasting reductions in alcohol consumption as well as alcohol abstinence [40]. Acamprosate has been shown to be safe and efficacious in promoting abstinence in patients recently detoxified [45, 79]. Since detoxified patients frequently show signs of depression, these medications can be enhanced in their efficacy with the use of adjunct antidepressant medications [80].

For those individuals who are detoxified from opioids and prefer a nonopioid maintenance treatment, both oral and extended-release naltrexone can be used [41, 81]. For these patients, the absence of physical dependence to opioids is required prior to receiving naltrexone. Oral naltrexone has been shown to be effective in relapse prevention with highly motivated patients or patients who have medication support structures; for patients receiving extended-release naltrexone studies have shown total confirmed abstinence during the treatment period and a significantly greater reduction in opioid craving and a significantly longer retention in care and treatment. Thus, extended-release naltrexone has been shown to be a useful treatment option for the prevention of relapse to opioid dependence, following opioid detoxification.

## 7. Use of Pharmacotherapies in the Treatment of Opioid Dependence in HIV Primary Care

The integration of treatment of substance-related and addictive disorders has a substantial impact on multiple HIV clinical outcomes including patient morbidity and mortality, adherence to antiretroviral treatment, quality of life, and HIV transmission [82]. Methadone maintenance treatment alone has been shown to reduce the rate of HIV infection in treatment cohorts by over 50% [83]. The introduction of medication assisted treatment in primary care has provided opportunities to integrate primary medical care for people living with HIV/AIDS with care and treatment for opioid dependence. Integrating primary medical care and medication assisted treatment using buprenorphine for opioid dependence can improve the health outcomes among people who use drugs because it provides an opportunity to address the health-related consequences of people living with HIV, particularly the health negative consequences of injection drug use [84, 85]. Multiple models have been piloted for the integration of medication assisted treatment using buprenorphine within HIV primary care [86, 87]. These include an on-site combination of addiction treatment/HIV specialist treatment; HIV primary care physicians prescribing buprenorphine; a nonphysician health care provider integrating medical care and substance abuse treatment services using buprenorphine; and community outreach model where buprenorphine is provided along with medical services in a mobile van or where buprenorphine is provided through a community-based recovery center. These service models have uncovered barriers to integrating medication assisted treatment using buprenorphine within HIV primary care that are both financial and regulatory. Regulatory challenges include licensing and training restrictions imposed by the Drug Addiction Treatment Act of 2000 and confidentiality regulations for alcohol and drug treatment records [88]. These models of care are important because buprenorphine has fewer adverse events associated with its use in patients and fewer drug-drug interactions among patients with HIV disease that require treatment with antiretroviral therapy [89, 90]. Also, buprenorphine is available by prescription. Thus, HIV treatment providers and primary care providers could offer both medication treatment for opioid dependence and concurrent treatment of HIV disease with an eye to drug-drug interactions between HIV medication and addiction pharmacotherapies. Therefore, based on these considerations, it may be preferable to utilize buprenorphine maintenance treatment rather than methadone maintenance treatment for patients with HIV disease and opioid dependence.

## 8. Use of Prescription Drug Monitoring Programs for Patient Care and Federal Programs that Support the Use of Prescription Drug Monitoring Programs

Prescription drug monitoring programs (PDMPs) often referred to as PDMPs or PMPs are state housed data systems that collect controlled substance prescription drug dispensing information as dictated by their state legislation. PDMPs were historically set up as law enforcement tools to monitor drug diversion and fraud but have evolved over the years to also serve as a clinical decision making tool to ensure the appropriateness of medication therapy as well as identifying individuals who may need further assessment or treatment.

Information typically available from a PDMP report include patient information (i.e., name, address, date of birth, gender, and patient identifier), prescriber information (i.e., name, address, DEA number, and phone number), pharmacy information (i.e., pharmacy name, address), and drug information (i.e., date drug was dispensed, drug quantity, drug strength, and source of payment in some states). However, there is some variability with what information is available because of the state laws that govern each of the PDMPs. Most states collect controlled schedules II–IV prescription medications and specific states collect nonscheduled drugs, such as tramadol. PDMPs are used by healthcare professionals, licensing and regulatory boards, law enforcement for drug investigations, state medical examiners or coroners, and/or research organizations for analysis and research [91].

Many primary care providers take advantage of existing systems that allow for delegates in some states to check PDMPs under the supervision of a physician. If that option is unavailable, providers may want to consider triaging and checking the PDMP only when prescribing controlled substances or when they suspect their patient is diverting, misusing, or abusing medications. Another option when limited by time is to run the PDMP reports for new patients or patients who travel long distances to attain treatment at their practice setting.

Important aspects of the report for the primary care provider are the drugs dispensed, quantity, dates, prescribers, and pharmacies, as well as determining suspicious patterns, if any. In a case study, a medical director from an opioid treatment program found that most of the patients were forthcoming in their medication history reports [92]. It was only a small number of patients who fell outside of the norm. Once the physician became familiar with their PDMP, it took about twenty seconds to check a patient's history and some additional time if they found prescriptions in the report. The study suggests informing patients that the PDMP is being monitored in advance rather than after the report is run so that patients-provider relationship is not damaged, since it is likely that not all of the patients will be receptive to the monitoring.

However, while the PDMP report offers an opportunity for conversations to take place about potential drug-drug interactions and therapeutic duplications, physicians have often expressed that this is a difficult conversation to have. Many primary care providers are untrained to deal with prescription drug misuse and abuse and according to a survey done in Oregon (http://www.acumentra.org/assets/PDMP-Presentation_Survey_Focus-Groups.pdf) are likely to discharge their patients or have their patient leave their practice “quietly” based on PDMP findings, instead of working with the patient to develop a comprehensive care plan for their addiction while continuing to serve as their primary care provider. Other options providers should consider including developing an ongoing relationship with an addiction specialist. The Oregon researchers conclude that there does not appear to be a need for training on how to use the PDMP but more importantly, training on how to respond to the information, especially resources on how to manage substance use disorders. Other barriers to accessing PDMP data include a lack of a seamless transaction that exists in the current clinical workflow, requiring the provider to log into one health information technology (HIT) system, only to turn around and access yet another one using a different user identification. Thus, despite the large repository of data in these systems, use in general has been reported to be suboptimal. The Department of Health and Human Services is supporting multiple programs to integrate PDMPs into HIT systems to allow for this valuable data source to be within reach to healthcare professionals. Instead of creating new systems, the thought is to use already existing HIT systems to connect with PDMPs and deliver this valuable information to healthcare professionals, such as prescribers and dispensers. The Office of the National Coordinator for Health Information Technology (ONC) along with SAMHSA supported a project called the Enhancing Access to PDMP using HIT to form workgroups to define barriers and recommend solutions to increase the use of PDMPs. This project also conducted pilots across the country to test the use of HIT to access PDMPs. The pilots allowed providers to receive certain critical information that was not available prior to their participation in the pilots. Some examples include having “person of interest” alerts provided on a weekly basis when “at risk” threshold of prescription drugs was met and integration of a patient's controlled substance prescription history information with prescription information available on e-prescribing software. Immediate improvement to patient care access was achieved and user workflows were streamlined and improved [93].

SAMHSA also supports programs that expand on the work done in the “Enhancing Access” project by providing funding to 17 states over two years to integrate their PDMPs in various HIT systems. The goal, like the Enhancing Access project, is to integrate PDMPs into three clinical settings: the provider practice, pharmacy, and emergency department.

When used optimally, PDMPs can be a useful clinical resource for primary care providers. Primary care providers not utilizing this resource miss an opportunity to consult with their patients to address a potential substance use disorder, discuss treatment services, or clarify a mistake taken place inadvertently by a pharmacy, or identify cases of identity theft important to the patient.

## Figures and Tables

**Table 1:** Pharmacotherapies in the treatment of substance use disorders.

Medication	Dosage form	Mechanism of action	DEA schedule	Application in primary care
Methadone	Tablet: 5 mg, 10 mg Tablet for suspension: 40 mg Oral concentrate: 10 mg/mL Oral solution: 5 mg/5 mL, 10 mg/5 mL Injection: 10 mg/mL	Mu agonist at the mu opioid receptor and also possible antagonist at the N-methyl-D-aspartate receptor	CII	Based on federal regulations primary care integration into/or linkage with Opioid Treatment Programs is required

Buprenorphine-naloxone	Sublingual film: buprenorphine/naloxone 2 mg/0.5 mg, 4 mg/1 mg, 8 mg/2 mg, and 12 mg/3 mg Sublingual tablet: buprenorphine/naloxone 1.4 mg/0.36 mg, 2 mg/0.5 mg, 5.7 mg/1.4 mg, and 8 mg/2 mg	Buprenorphine: Partial mu agonist at the mu opioid receptor and an antagonist at the kappa opioid receptor Naloxone: antagonist at mu opioid receptor and produces withdrawal signs/symptoms	CIII	To prescribe buprenorphine in a primary care setting, a physician must obtain a waiver from SAMHSA and be issued an additional registration number by the DEA (see http://www.dpt.samhsa.gov/ for details)

Buprenorphine		Partial mu agonist at the mu opioid receptor and an antagonist at the kappa opioid receptor	CIII	Buprenorphine without naloxone products are indicated only for patients with documented hypersensitivity to naloxone

Naltrexone	Tablets: 25 mg, 50 mg, and 100 mg Extended-release injectable suspension: 380 mg/vial	Opioid antagonist with highest affinity for the mu opioid receptor Little or no opioid agonist activity Produces some pupillary constriction by an unknown mechanism	n/a	Provided by prescription; naltrexone provides a blockade of opioid receptors, reduces cravings, and diminishes the rewarding effects of alcohol and opioids Extended-release injectable naltrexone is indicated for the prevention of relapse to opioids or alcohol

Acamprosate	Delayed-release tablet: 333 mg	Mechanism not completely understood; studies suggest that acamprosate may interact with glutamate and GABA neurotransmitter systems centrally	n/a	Provided by prescription; acamprosate reduces symptoms of protracted abstinence associated with chronic alcohol exposure and alcohol withdrawal

Disulfiram	Tablet: 250 mg, 500 mg	Blocks oxidation of alcohol at the acetaldehyde stage	n/a	When taken in combination with alcohol, disulfiram causes severe physical reactions, including nausea, flushing, and heart palpitations The knowledge that such a reaction is likely if alcohol is consumed acts as a deterrent to drinking

Naloxone	Injection: 0.4 mg/mL, 1 mg/mL, and 0.4 mg/0.4 mg	Opioid antagonist; in vitro studies suggest that it antagonizes opioid effects by competing for the mu, kappa, and sigma opiate receptor sites in the CNS, with the greatest affinity for the mu receptor	n/a	Naloxone is the antidote to opioid toxicity It reverses the respiratory depression induced by opioids It has no psychoactive properties and no abuse potential To be effective naloxone should be administered as soon as possible when opioid overdose is suspected, usually prior to transport to an emergency department Because of this naloxone should be prescribed to persons at risk of opioid overdose for emergency administration Education and training should be provided to the patient and family members on how to reduce risk, recognize, and respond to overdose appropriately Naloxone is typically dispensed in an injectable form and may require prescription of syringes

*Note*. This table highlights some of the properties of each medication. It does not provide complete information and is not intended as a substitute for the package inserts or other drug reference sources used by clinicians. For patient information about these and other drugs, the National Library of Medicine provides MedlinePlus (http://medlineplus.gov/). Adapted from the National Institute on Alcohol Abuse and Alcoholism (NIAAA). Helping Patients Who Drink Too Much: A Clinician's Guide. Updated 2005 Edition (NIH Publication number 07-3769). Bethesda, MD: NIAAA, National Institutes of Health, 2007; Substance Abuse and Mental Health Services Administration. 2013; and Alkermes, Inc. Medication Guide. Revised November 2010 (VIV993B). Waltham, MA: Author, 2010a (Accessed at http://www.vivitrol.com/Content/pdf/medication_guide.pdf).
